# Gout: will the “King of Diseases” be the first rheumatic disease to be cured?

**DOI:** 10.1186/s12916-016-0732-1

**Published:** 2016-11-11

**Authors:** Jasvinder A. Singh

**Affiliations:** 1 Birmingham VA Medical Center, Birmingham, AL USA; 2 Department of Medicine at the School of Medicine, University of Alabama at Birmingham, Birmingham, AL USA; 3 Division of Epidemiology at the School of Public Health, University of Alabama at Birmingham, Birmingham, AL USA

**Keywords:** Gout, Urate-lowering therapy, Serum urate, Lesinurad, Allopurinol

## Abstract

Gout is the most common inflammatory arthritis in adults in the Western world. Characterized by hyperuricemia and the effects of acute and chronic inflammation in joints and bursa, gout leads to an agonizing, chronically painful arthritis. Arthritis can also be accompanied by urate nephropathy and subcutaneous urate deposits (tophi). Exciting new developments in the last decade have brought back the focus on this interesting, crystal-induced chronic inflammatory condition. New insights include the role of NALP3 inflammasome-induced inflammation in acute gout, the characterization of diagnostic signs on ultrasound and dual-energy computed tomography imaging modalities, the recognition of target serum urate less than 6 mg/day as the goal for urate-lowering therapies, and evidence-based treatment guidelines. A better understanding of disease mechanisms has enabled drug discovery – three new urate-lowering drugs have been approved in the last decade, with several more in the pipeline. We now recognize the important role that environment and genetics play in the causation of gout. A focus on the cardiac, renal, and metabolic comorbidities of gout will help translational research and discovery over the next decade.

## Editorial

Gout is the most common inflammatory arthritis in the USA and other Western countries [1–3]. Despite being four times more prevalent than its autoimmune counterpart, rheumatoid arthritis (RA), it lags far behind in the number of publications on the topic (15,475 vs. 129,452 in PubMed search using terms “rheumatoid arthritis” vs. “gout or gouty arthritis” on 10/9/2016, i.e., by approximately one-tenth), partially reflecting the interest it traditionally generated from the researchers, pharmaceutical companies and the federal funding agencies.

Gout is one of the oldest diseases described in humans and is often considered an “old disease” [4]. So, why has gout not proven as “popular” as RA among researchers and clinicians in the past? Is it because gout is not as enigmatic as an autoimmune arthritis such as RA? Is it due to the fact that we have had definitive effective inexpensive treatment options (allopurinol, probenecid etc.) available for gout (albeit not effectively used) since the 1960s and 70s? Is it because gout symptoms are intermittent, at least in the initial phase of the disease? Is it due to the recognition that behaviors such as overconsumption of certain foods (including red meat and alcohol) and associated obesity are risk factors for gout? I remain unsure about the reasons behind gout’s lack of appeal, but the past seems to be the past; things are changing very rapidly in the world of gout. For example, the number of gout publications have increased 2.5-fold from 290 in 2005 to 753 in 2015 in PubMed (search 10/9/2016). Many exciting developments in gout, including new drug discoveries, have occurred in recent years and the field continues to evolve at a dramatic pace. Novel disease mechanisms have been uncovered and new knowledge with the potential to change our understanding of inflammation and how it can affect different body systems has emerged. The sections below provide a snapshot of some of the key developments.

### Gout as an inflammatory disease

The link between inflammasome and associated inflammation in gout is now well understood [5–7]. The NALP3 (also called cryopyrin) inflammasome complex is a key regulator of the innate inflammatory phenotype of several diseases, including gout and type 2 diabetes [5]. Martinon et al. [6] showed that (1) monosodium urate crystals engaged caspase-1, leading to NALP3 activation and to an increase in active interleukin (IL)-1b and IL-18 production; (2) induced macrophages from mice deficient in various inflammasome components, such as caspase-1, ASC, and NALP3, were defective in urate crystal-induced activation of IL-1b; and (3) an impaired neutrophil influx was also found in an in vivo model of crystal-induced peritonitis in inflammasome-deficient mice or mice deficient in the IL-1b receptor. Additional evidence of the role of IL-1 in acute inflammation in gout was shown in a murine model of gout, where inflammation following monosodium urate injection into the mouse ankle joint was significantly reduced both in mice deficient for the IL-1 receptor and in wild type mice treated with the IL-1 inhibitor IL-1 Trap (rilonacept) [8, 9]. Clinical studies showed that treatment with medications targeting anti-IL-1 (IL-1RA [anakinra], IL-1Trap, and anti-IL-1β monoclonal antibody [canakinumab]) were each associated with a rapid response in patients with acute gouty arthritis, thereby reinforcing the argument for an important role for IL-1β in gout pathogenesis [10–12].

### New classification criteria and imaging in gout

In a collaborative effort, the American College of Rheumatology (ACR) and the European League against Rheumatism (EULAR) developed the new 2015 classification criteria for gout [13]. It is a scoring system based on a combination of clinical features, signs, and symptoms, in combination with radiographic, and ultrasound, computed tomography (CT), or biochemical findings (each criteria scored from -4 to 4). The presence of urate crystals on polarized microscopy, or in its absence, a total score of ≥ 8, classifies an individual as having gout [13]. The sensitivity and specificity of these new criteria were at 92 % and 89 %, respectively. These classification criteria should help with clinical trials and prospective cohort studies in gout. Nevertheless, their utility in database and retrospective studies remains to be seen, given the specificity of the clinical signs and symptoms, and paucity of such data in clinical records and databases.

The role of imaging in gout has expanded tremendously in recent years, especially with the introduction and increasing use of ultrasound and dual-energy CT (DECT) in clinical practice. While many ultrasound features of gout have been described, two findings that are considered pathognomonic include the presence of double contour sign or the starry sky appearance, caused by urate crystal deposits on cartilage surfaces (cartilage enhancement presenting as a parallel line to bony articular surface) versus within the joint fluid, respectively [14, 15]. DECT is a non-invasive, sensitive, and reproducible method of identifying urate deposits in joints and in periarticular tissue by allowing a simultaneous direct visualization of urate deposits and bone structures using different display colors [16]. The attenuation of urate differs significantly from that of bone, depending on the kilovolt setting of the X-ray tube. However, other crystalline diseases, such as calcium pyrophosphate deposition, can lead to an ultrasound appearance of double contour similar to that of gout [17]; therefore, it is now arguable whether double contour is specific for gout or for crystalline arthritis [18]. These modalities are providing insights into a better understanding of disease pathology and pathophysiology.

### Treatment guidelines, treat-to-target serum urate, and new drugs for gout

Treatment guidelines for gout have been recently published by both the ACR in 2012 [19, 20] and the EULAR in 2016 [21]. Several key aspects of appropriate management are addressed in these treatment guidelines, which should be helpful to providers since gout care is critically suboptimal [22]. A controversial recommendation to limit the maximum dose of allopurinol in patients with gout and chronic kidney disease by adjusting to creatinine clearance in EULAR guidelines [21] has been challenged [23] since the risk of hypersensitivity reactions associated with allopurinol seems to be related to the starting dose, not the maximal dose [24]. Therefore, there is currently no rationale to limit the maximum dose of allopurinol in patients with gout and chronic kidney disease.

Treat-to-target (T2T) serum urate (sUA) is not a new concept in gout, but one that has been brought to center stage by the leading treatment recommendations in the ACR [19] and the EULAR guidelines [21], as well as by a recent consensus statement about T2T [25]. The rheumatology community considers the existing evidence regarding T2T to a goal of sUA less than 6 mg/dL in gout as sufficient based on three key correlates for achieving sUA less than 6 mg/dL, namely (1) associated benefits of reduction of gout flares, tophi, and medical care costs by achieving and maintaining this target [26–28]; (2) the fact that this sUA target is below the solubility threshold of urate, which prevents its crystallization in body fluids at 6.8 mg/dL; and (3) the use of this sUA threshold as a primary outcome in gout randomized controlled trials (RCTs) for the drug approval of urate-lowering therapies (ULTs) by the regulatory authorities [26, 29, 30]. Since there is no way to achieve the sUA target without monitoring or reassessing sUA, rheumatologists monitor sUA and aim for an sUA target level of less than 6 mg/dL. Therapeutic doses of allopurinol (100–800 mg/day) or febuxostat (40–80 mg/day) or a combination with uricosurics is often needed to achieve target sUA. Allopurinol maximum dose need not be reduced, even in the presence of renal failure, since adverse events are related to initial, and not final, allopurinol dose [24]; pegloticase is another option. Appropriately titrated ULT doses can help achieve a near cure for gout by resolving all urate crystals.

On the other hand, the Agency for Healthcare Research and Quality determined that the evidence that sUA monitoring in patients with gout improves outcomes was insufficient, and that the lowering of sUA below a threshold had low level of evidence due to the absence of a randomized trial testing this strategy [31]. However, evidence to the contrary has been available as of 2005 and 2011 [30]. In two replicate pivotal studies of pegloticase, a uricase that lowers sUA, both the pegloticase biweekly (currently approved by the US FDA and used in clinical practice) and monthly dose groups had a higher rate of responders (defined as patient with plasma UA less than 6.0 mg/dL for 80 % of the time or longer during both months 3 and 6), at 38–47 % and 20–49 %, respectively, versus 0 % in the placebo group [30]. Further, complete resolution of one or more tophi at the final visit was clinically meaningfully and statistically significantly higher in both pegloticase dose groups (biweekly and monthly dosing 40 % and 21 %, respectively, vs. 7 % in placebo) [30]. In the 12-month pivotal febuxostat active comparator RCT, the respective median percent reduction in the tophus area by week 52 for subjects receiving febuxostat 80 mg or 120 mg were 83 % and 66 %, respectively, versus 50 % in the allopurinol 300 mg daily group, in synchrony with the proportion of patients who achieved target sUA less than 6 mg/dL at the last 3 monthly visits (53 % and 62 % vs. 21 %, respectively) [29]. Thus, achieving target sUA < 6 mg/dl with effective ULT in randomized trials was associated with better gout outcomes , i.e., reduction in tophi size and tophi resolution.

Two new ULTs, febuxostat and pegloticase, were approved in the last decade in several countries, including the USA and the European Union. Importantly, data on two new drugs have recently been published. The pipeline for gout treatments looks very promising [32]. One of them, lesinurad, is now approved for use in the USA and the European Union [33, 34]. Lesinurad is a selective inhibitor of urate/anion exchanger 1 (URAT1) and organic acid transporter 4 (OAT4), two urate transporters responsible for the reabsorption of urate from the proximal renal tubule [35], making it one of the newest approved ULTs. One of two replicate studies, Combining Lesinurad With Allopurinol in Inadequate Responders-1 (CLEAR-1 in the US [36], with CLEAR-2 completed in Europe but not yet published), showed that 54.2 % of patients in the lesinurad 200 mg plus allopurinol group and 59.2 % in the lesinurad 400 mg plus allopurinol group, versus 27.9 % in the placebo plus allopurinol arms achieved the primary trial end-point of sUA less than 6 mg/dL at 6-months, with the differences being statistically significantly different from placebo. Renal function elevations were noted in the 200 mg group, but with a greater frequency in the 400 mg group, and the US FDA approved the 200 mg lesinurad dose in combination with allopurinol for patients refractory to allopurinol. The second drug is arhalofenate, which has a dual action and inhibits urate transporter URAT-1 and proinflammatory cytokines, including IL-1b. This drug is not yet approved for use. In a RCT, 239 gout patients were randomized, respectively assigned at a 2:2:2:2:1 ratio to receive 600 mg arhalofenate, 800 mg arhalofenate, 300 mg allopurinol, 300 mg allopurinol plus 0.6 mg colchicine, or placebo once a day [37]. Gout flares were significantly reduced with 800 mg arhalofenate versus 300 mg allopurinol, with a 46 % decrease in the 800 mg arhalofenate group (0.66 vs. 1.24 (*P* = 0.006) and vs. placebo (*P* = 0.049)) [37]. Several other treatments with great potential as ULTs or for acute flares are currently under development [32].

### The comorbidities of gout and hyperuricemia

The association of gout with comorbidities has long been known [38–40]. In the US National Health and Nutrition Survey 2007–8 [40], 74 % and 71 % of patients with gout reported a physician diagnosis of hypertension and chronic kidney disease stage 2 or higher. Diabetes and nephrolithiasis are common comorbidities in patients with gout, with a prevalence of approximately 25 %, and heart failure, myocardial infarction, and stroke are also commonly observed. New data now emerging indicate the potential benefit of lowering sUA on non-arthritic comorbidity load in gout. Many have speculated on the cardiovascular benefits of sUA lowering [40, 41], but interest in its reno-protective effect is also emerging [42]. An ongoing $24.3 million NIH-funded study will compare allopurinol to placebo in delaying or preventing early nephropathy in type 1 diabetics without gout [43]. Other studies assessing benefits of sUA lowering in patients with hyperuricemia and chronic kidney disease stage 3 are also underway [44].

### Future directions

The future of gout is bright. The pipeline for discovery looks very promising for new therapies for acute gout and urate lowering. Personalized medicine for gout may be just around the corner, as our understanding of the role of genetics and environment improves. While our knowledge of disease mechanisms in gout has improved dramatically, the quality of care remains suboptimal and under-treatment is common. I see an excellent future for the disease if efforts within the next decade are focused on a three-pronged approach encompassing (1) the appropriate use of existing effective therapies with allopurinol as the key example, as well as others; (2) novel discovery and approval of new medications for acute gout and long-term urate-lowering; and (3) a better understanding of the role hyperuricemia and chronic inflammation play in the occurrence of cardiac, renal, and metabolic comorbidities of gout in order to effectively improve the capacity to prevent and treat these comorbidities.

## Conclusion

In summary, several new developments in gout, including the recognition of the role that innate immunity plays in crystal-induced inflammation in gout via activation of NALP3 inflammasome, the implementation of T2T sUA less than 6 mg/dL as an important goal relevant to patients, and new imaging techniques, classification criteria, and treatment guidelines, all provide a positive outlook for the treatment of the disease. The launch of new medications for treatment and a robust pipeline add to the new opportunities in optimizing gout treatment. The associated cardiovascular and renal comorbidities and the potential benefit of ULTs on these outcomes identify another important aspect of gout and its treatment. Gout, which has long been a disease of high interest for rheumatologists and clinical immunologists, should now be noticed by internists and family and general practitioners. I hope that the federal and other funding agencies become aware of the transformation of this old disease into an opportunity to learn about inflammation pathways and their impact on the associated comorbidities.

In recognition of the exciting developments in this field, *BMC Medicine* and *BMC Musculoskeletal Disorders* are launching an article collection focusing on the research that provides important developments in gout management and therapy. Authors interested in submitting to the collection are advised to visit the following link: http://bmcmedicine.biomedcentral.com/articles/collections/gout.

## Abbreviations

ACR: American College of Rheumatology
EULAR: European League against Rheumatism
IL: Interleukin
RA: Rheumatoid arthritis
sUA: Serum urate
T2T: Treat-to-target
ULT: Urate lowering therapy
